# Patient and caregiver motivators and barriers to eczema clinical trial participation: Analysis of survey data

**DOI:** 10.1002/ski2.259

**Published:** 2023-06-22

**Authors:** Michael Evan Jacobson, Isabelle J. Thibau, Wenelia Baghoomian, Emile Latour, Ajai Kastala, Allison R. Loiselle, Eric Lawrence Simpson, Wendy Smith Begolka

**Affiliations:** ^1^ Department of Dermatology Oregon Health & Science University Portland Oregon USA; ^2^ National Eczema Association Novato California USA; ^3^ Biostatistics Shared Resource Knight Cancer Institute Oregon Health & Science University Portland Oregon USA

## Abstract

**Background:**

Eczema clinical trials (CTs) are increasing in number, yet participation across the eczema community is low. Little is known about patient characteristics and views on motivators and barriers to CT participation (CTP).

**Objectives:**

Determine factors that motivate or impede participation in eczema CT and respondent characteristics associated with these factors.

**Methods:**

Qualitative thematic analysis was performed on open‐ended questions from an online survey that collected respondent demographics, understanding of and experience with CTs, and drivers/barriers to CTP. Mixed‐methods analysis included 924 respondents, 728 (78.8%) adults with eczema and 196 (21.2%) caregivers of children with eczema.

**Results:**

A large proportion (71.8%) of respondents would potentially participate in CTs. The most common theme for why a respondent considered or would explore CTP was burden of disease (81.0% and 57.3% respectively). Among those who participated in or considered a CT, caregivers (*p* = 0.001) reported fewer altruistic motivations compared to adult patients, with trends towards men citing disease burden more (57.0% vs. 50.9%) and altruism less (14.5% vs. 19.2%) than women. Lack of awareness (57.7%) was the most common reason for never having considered a CT. Among those who never considered CTP, age (*p* = 0.012) and eczema severity at its worst (*p* = 0.002) were associated with reasons why they never participated. Specifically, older and less severe patients had greater perceptions of eligibility as a barrier to CTP. Caregivers more commonly cited fear of CT risks (20% vs. 11.4%) compared to adult patients who cited accessibility concerns (17.7% vs. 8.6%) as barriers to CT exploration. A subgroup of respondents that never considered CTP and extremely unlikely to consider CTs cited more fears/risks/unknowns and accessibility barriers to CTP. No significant differences in motivators or barriers were observed across race/ethnic groups and urban/rural populations.

**Conclusions:**

Motivating factors for CTP include greater disease burden; lack of awareness represents a large barrier. Healthcare providers are trusted intermediaries with ability to refer and inform about CTs; they have a potentially significant role in raising awareness and discussing eczema patient/caregiver perspectives related to CTP. Investigators should tailor recruitment approaches and study design where possible to address identified motivators and barriers.

## Graphical abstract

This study from the US by the National Eczema Association aimed to determine which demographic and clinical characteristics contribute to eczema patient/caregiver motivation (or lack thereof) to participate in CTs, issues getting in/staying in a trial, and likelihood to consider participating in the future. Burden of disease was the biggest motivator for participating among both those who had (81%) and hadn't (57%) considered participating before; lack of awareness was the biggest barrier (58%). Caregivers cited altruism less than adult patients as a reason for having considered CTs. Older age and those with less severe eczema cited eligibility concerns were preventing them from considering CTs. No differences were observed based on race/ethnicity or urban/rural location.

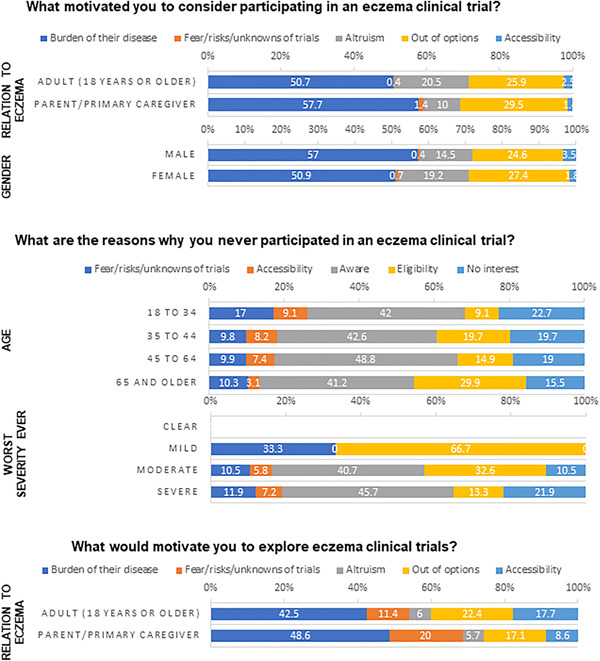

**What is already known about this topic?**

- Participant recruitment is a leading barrier to successful clinical research, particularly interventional clinical trials (CTs). Despite a rapid increase in the number of eczema clinical trials being initiated, little is known of what motivates and hinders clinical trial participation (CTP) from the patient and caregiver guardian perspective.

**What does this study add?**

- Our study identified a complex landscape of motivators and barriers to trial participation. Disease burden was a strong motivator in participants who considered and would explore trial participation. Caregivers cited altruism less as a reason for having considered CTs than adult patients. Older populations and those with less severe eczema cited perceived study eligibility concerns preventing them from considering CTs. No differences were observed based on race/ethnicity and urban/rural demographics.

**What are the clinical implications of this work?**

- With the emergence of new eczema therapies and increase in the number of clinical trials, the risk of poor accrual and need for diverse populations to ensure generalisability are ever greater. Our work provides the most thorough description of the different patient‐ and caregiver‐reported motivators and barriers and the importance of dispelling myths and misconceptions of CTP, specifically eligibility. Stakeholders can use this information to optimise recruitment in eczema CTs.

## 1 INTRODUCTION

Clinical trials (CTs) play a critical role in modern medicine by evaluating new treatments and generating evidence to inform clinical practice. Participants can also benefit from CTs by accessing diverse opportunities such as education or new and/or otherwise unavailable treatments. However, these benefits can only be realized if CTs achieve sufficient recruitment to accomplish their goals. This is particularly relevant for eczema, a condition which encompasses seven skin diseases characterised by itchy, inflamed skin and is estimated to affect over 31 million Americans and 1 in 10 individuals worldwide.^1^ ^−^ ^6^ The pipeline for eczema treatments is extremely active and eczema CTs are also increasing.^7^, ^8^, ^9^ A recent estimate of newly initiated CTs registered on ClinicalTrials.gov showed a 3.3‐fold increase since 2008. However, a survey of 1508 patients with eczema and caregivers showed that just 8% had or were currently participating in CTs.^10^ Participant recruitment challenges in eczema CTs could jeopardise drug development and poor enrolment may yield underpowered studies, which can negatively affect the validity of results or cause changes in trial methodology, resulting in poor‐quality evidence and limit patient outcomes.^11^, ^12^, ^13^ High CT participation (CTP) and better representation from diverse populations and eczema experiences are important to evaluate these potential treatment options and improve generalisability.

Few studies have investigated barriers and motivators to CTP. In a single institution survey of adults and parents of children with AD, very few (10.2% and 9.4% respectively) reported being offered the opportunity to participate in clinical research for eczema.^14^ Respondents who had participated in research cited motivators including the benefit to other patients, contributing to knowledge advancement, and their feeling that the study's objectives were important.^14^ A more recent survey from the National Eczema Association (NEA) of patients with eczema found that knowledge of terms associated with CTs was associated with both interest and past participation in CTs. The study further reported that individuals with greater confidence in their ability to acquire CT information were more likely to be interested in participation.^15^ The rise of CTs and the current lack of information on barriers and motivators to eczema CTP urge the generation of new knowledge for the optimization of CT participant recruitment.

In this exploratory study we aim to determine which personal demographic and clinical factors contribute to eczema patients' and caregivers' motivation (or lack thereof) to CTP, issues getting in or staying in a trial, and their likelihood to consider participating in the future.

## 2 PATIENTS AND METHODS

### 2.1 Study design and population

From 1 May to 6 June 2020, a 46‐question online survey was administered to United States (US) and US territory residents who were adult patients (age ≥18 years) and caregivers of children (age 0–17 years) with eczema. Eczema was defined as one or more of the following self‐reported diagnoses: atopic dermatitis, allergic/irritant contact dermatitis, dyshidrotic eczema, hand eczema, neurodermatitis, nummular eczema, seborrhoeic dermatitis, and/or stasis dermatitis. Using a voluntary response convenience sampling strategy, the survey was advertised on NEA platforms via NEA website, email, and social media to reach those beyond the NEA email list to address sampling bias. Respondents were screened for eligibility and informed consent was obtained. Duplicate entries were avoided by preventing access to the survey twice. No remuneration was offered for participation, however respondents who completed the survey were entered into a drawing to win one of ten $50 Amazon.com e‐gift cards. This study was deemed exempt from full review by the Western Institutional Review Board‐Copernicus Group. NEA researchers developed the survey and patients and caregiver volunteers were involved in pilot testing and providing feedback on content and literacy level before the survey was finalised.

A subset of the survey, which formed the basis of this work, collected information on respondent demographics, understanding of and experience with CTs, and drivers for and barriers to CTP. Based on previous CT experience, participants were directed to respond to open‐ended questions detailing their motivations (or lack thereof) and barriers (if applicable) for considering CTP. Those with no previous experience and who had never considered CTP described reasons why they never participated in CTs and what would motivate them to explore CT opportunities. Those who had considered CTP with/without attempt to enrol described what motivated them to consider participating and any difficulties enrolling in CTs. A subgroup of these participants who had previous CTP additionally described any difficulties staying in a trial. All participants were asked a final question rating their likelihood to consider participating in an eczema CT (answer options: extremely likely, somewhat likely, neutral (neither likely nor unlikely), somewhat unlikely, and extremely unlikely).

### 2.2 Qualitative analysis

Using qualitative analysis, open‐ended responses were blind coded with the intent of identifying constructs in the text using words or phrases to substantiate and then analysed for overarching themes. A set of response codes for each question was developed from each of two evaluators (W. Baghoomian and M. Jacobson; M. Jacobson and A. Kastala) on a first pass using just 25% of responses. All responses were then coded using the set developed in the first pass and disagreements between the two evaluators were resolved by a blinded third evaluator (I. Thibau; W. Baghoomian). Multiple codes could be attributed to an open‐ended response if the respondent cited multiple reasons. Lastly, response codes were grouped into major themes for analysis. Responses not answering the question were excluded from analysis.

### 2.3 Statistical analysis

Statistical analysis was based on interpretive and statistical approaches to identify similar patterns and themes and then seek out commonalities and differences among the data to extract for further consideration and analysis.

Geographic location was defined as Urban/Suburban versus Rural using Rural‐Urban Continuum Codes (RUCC) (Urban/Suburban [RUCC 1–3], Rural [RUCC 4–10]).^16^ As respondents were allowed to report multiple eczema diagnoses, eczema severity was determined by selecting the diagnosis with the worst patient/caregiver‐reported global severity.

Descriptive statistics were used to summarise demographic characteristics and major themes from survey respondent responses (means and standard deviations for continuous variables; frequencies and percentages for categorical variables). Group comparisons for categorical variables were performed using Fisher's Exact test; group comparisons with one‐way ANOVA for continuous variables. In the cases where multiple codes could be selected from one open‐ended response (multiple response categorical variable), tests for simultaneous pairwise marginal independence were used with the R package, MRCV.^17^, ^18^ The analysis was performed using R: A Language and Environment for Statistical Computing.^19^ Statistical significance was set at *p* < 0.05. Bonferroni corrections were used to adjust for multiple comparisons in post‐hoc analysis.

## 3 RESULTS

### 3.1 Respondents, respondent demographics, and patient health characteristics

A total of 1285 adult eczema patients and caregivers of children ages 0–17 with eczema participated in the survey, of which 361 did not respond to any open‐ended questions and were excluded. Analysis was therefore based on 924 respondents (94.2% completion rate), 728 (78.8%) adults with eczema and 196 (21.2%) caregivers of children with eczema. Respondent demographics are presented in Table 1. At the time of survey response, 27.3% reported their/their child's eczema as severe, 43.4% moderate, 24.6% mild, and 4.8% clear. Most respondents (80.7%) reported their/their child's worst experienced severity of any eczema diagnosis as severe. Previous CTP was reported by 10.4% (7.6%‐1 trial, 1.4%‐2 trials, 1.4%‐3 or more trials), 15.5% had no previous CTP but had considered participation and attempted to do so, 43.7% had no previous CTP but had considered participation without attempt to enrol, and 30.4% had no previous CTP and had not considered participation. Of the 870 participants who indicated their likelihood to consider future CTP, 69 (7.9%) were extremely unlikely, 75 (8.6%) somewhat unlikely, 101 (11/6%) neither likely nor unlikely, 321 (36.9%) somewhat likely, and 304 (34.9%) extremely likely.

**TABLE 1:** Respondent characteristics.

Category	Level	Number not missing	Overall	Adult (18 years or older)	Parent/primary caregiver	*p*
*n*			924	728	196	
Respondent age, continuous (mean (SD))		924	47.58 (17.28)	49.50 (18.51)	40.45 (8.48)	<0.001
Age, categorical (%)		924				<0.001
	18 to 34		252 (27.3)	209 (28.7)	43 (21.9)	
	35 to 44		182 (19.7)	85 (11.7)	97 (49.5)	
	45 to 64		311 (33.7)	257 (35.3)	54 (27.6)	
	65 and older		179 (19.4)	177 (24.3)	2 (1.0)	
Respondent gender (%)		924				<0.001
	Male		251 (27.2)	159 (21.8)	92 (46.9)	
	Female		667 (72.2)	564 (77.5)	103 (52.6)	
	Non‐binary		5 (0.5)	4 (0.5)	1 (0.5)	
	Other		1 (0.1)	1 (0.1)	0 (0.0)	
Respondent race (%)		912				<0.001
	White		658 (72.1)	544 (75.7)	114 (59.1)	
	Black or African American		85 (9.3)	53 (7.4)	32 (16.6)	
	American Indian or Alaskan Native		8 (0.9)	3 (0.4)	5 (2.6)	
	Native Hawaiian or Pacific Islander		4 (0.4)	3 (0.4)	1 (0.5)	
	Asian or Asian American		83 (9.1)	66 (9.2)	17 (8.8)	
	Some other race or ethnicity		29 (3.2)	25 (3.5)	4 (2.1)	
	Multiracial		45 (4.9)	25 (3.5)	20 (10.4)	
	I don't know/prefer not to answer		0 (0.0)	0 (0.0)	0 (0.0)	
Respondent ethnicity (%)		924				<0.001
	Hispanic		98 (10.6)	62 (8.5)	36 (18.4)	
	Not Hispanic		826 (89.4)	666 (91.5)	160 (81.6)	
Respondent race × ethnicity (%)		924				<0.001
	Hispanic or Latino		98 (10.6)	62 (8.5)	36 (18.4)	
	Not Hispanic or Latino, White		605 (65.5)	511 (70.2)	94 (48.0)	
	Not Hispanic or Latino, Black		83 (9.0)	53 (7.3)	30 (15.3)	
	Not Hispanic or Latino, Asian		81 (8.8)	64 (8.8)	17 (8.7)	
	Not Hispanic or Latino, other		57 (6.2)	38 (5.2)	19 (9.7)	
Respondent RUCA (%)		924				0.781
	Urban (1 to 3)		823 (89.1)	650 (89.3)	173 (88.3)	
	Rural (4 to 10)		101 (10.9)	78 (10.7)	23 (11.7)	
Patient diagnosed with by a healthcare provider with atopic dermatitis (%)		924				0.002
	0		132 (14.3)	118 (16.2)	14 (7.1)	
	Diagnosed with AD		792 (85.7)	610 (83.8)	182 (92.9)	
Patient worst severity (all types) (%)		924				0.201
	Clear		0 (0.0)	0 (0.0)	0 (0.0)	
	Mild		10 (1.1)	10 (1.4)	0 (0.0)	
	Moderate		168 (18.2)	128 (17.6)	40 (20.4)	
	Severe		746 (80.7)	590 (81.0)	156 (79.6)	
Patient worst severity now (all types) (%)		924				0.459
	Clear		44 (4.8)	36 (4.9)	8 (4.1)	
	Mild		227 (24.6)	185 (25.4)	42 (21.4)	
	Moderate		401 (43.4)	316 (43.4)	85 (43.4)	
	Severe		252 (27.3)	191 (26.2)	61 (31.1)	
Respondent previous CT participation/attempts/consideration (%)		924				0.762
	1		70 (7.6)	54 (7.4)	16 (8.2)	
	2		13 (1.4)	10 (1.4)	3 (1.5)	
	3 or more		13 (1.4)	10 (1.4)	3 (1.5)	
	None, but considered (and tried)		143 (15.5)	111 (15.2)	32 (16.3)	
	None, but considered (never tried)		404 (43.7)	313 (43.0)	91 (46.4)	
	None, never considered		281 (30.4)	230 (31.6)	51 (26.0)	
Respondent likelihood to consider (%)		870				0.386
	Extremely unlikely		69 (7.9)	59 (8.6)	10 (5.5)	
	Somewhat unlikely		75 (8.6)	63 (9.2)	12 (6.6)	
	Neither likely nor unlikely		101 (11.6)	81 (11.8)	20 (10.9)	
	Somewhat likely		321 (36.9)	246 (35.8)	75 (41.0)	
	Extremely likely		304 (34.9)	238 (34.6)	66 (36.1)	

### 3.2 Responses to open‐ended questions: Motivators

Using free text fields, respondents elaborated on motivators and, if applicable, barriers to participation. In questions aiming to understand motivation, themes relating to disease burden predominated, accounting for 81.0% of all “what motivated you to consider CTP” responses and 57.3% of all “what would motivate you to explore CTP” responses. Perception of being out of alternative options was the second most prominent motivator, responsible for 41.9% and 28.1% respectively to these two questions. The third most prominent motivator identified for having considered CTP was altruism (28.0%), although it was the least common response (7.8%) to the exploring CTP question; accessibility was the third most prominent motivator to explore CTP (21.0%).

### 3.3 Responses to open‐ended questions: Barriers

Respondents identified several barriers to CTP. Respondents' most reported barrier themes included lack of awareness (57.7% of all “why did you never participate in CTs” responses), CT eligibility requirements (49.4% of all “what were some of the issues getting in” responses; 23.8% of “why did you never participate in CTs” responses), and burden of CT participation/accessibility (64.7% of all “issues staying in” responses; 40.4% of “what were some of the issues getting in” responses). A lack of interest in CTP was cited by about a quarter (24.9%) of “why did you never participate in CTs” responses. Full response data are presented in Table 2.

**TABLE 2:** Open‐ended survey questions assessing motivation and barriers to clinical trial participation and the major themes described.

Major theme	Codes within major theme	Frequency major theme is present in open‐ended question (%)
*Shown to participants with previous eczema clinical trial participation or that had considered eczema clinical trial participation with/without attempt*		
**What motivated you to consider participating in an eczema clinical trial?** 987 major themes in 642 responses		
Burden of disease	Eczema controlled, expensive medication, self‐esteem, treat eczema self/Child	520 (81.0)
Out of options/out of control	Access otherwise unavailable care, didn't like current treatment options, frustration/desperation, no other option, wants other treatment options	269 (41.9)
Altruism	Curiosity/interest in research, curious about experimental treatment, develop new treatments	180 (28.0)
Accessibility	Compensation/money, physician recommendation	22 (3.4)
Fear/risks/unknowns of trials	Afraid of risks associated with clinical trials, doesn't understand clinical trials	7 (1.1)
**What were some of the issues getting in/enrolling in the eczema clinical trial?** 169 major themes in 135 responses		
Eligibility	Did not meet eligibility requirements: Age, other, severity, pregnancy status	67 (49.3)
Burden of trial/accessibility	Financial issues: Cost of participation, transportation, insurance, trial participation too burdensome: Time commitment, washout, other	55 (40.4)
Administrative	Administration issues‐investigator failed to recruit, unable to participate due to recruitment closure	27 (19.9)
Other	Unable to participate for no specified reason	15 (11.0)
Fear/risks/unknowns of trial	Fear of experimental, other reasons, side effects	6 (4.4)
*Shown only to subgroup of participants with previous eczema clinical trial participation*		
**What were some of the issues staying in the eczema clinical trial?** 23 major themes in 18 responses		
Burden/accessibility	Trial participation too burdensome/visit amounts, study procedures, scheduling conflicts, general	11 (64.7)
Negative experience	Negative treatment side effects, condition worsened, researcher pressure	9 (52.9)
*Shown to participants with no previous experience and who had never considered eczema clinical trial participation*		
**What are the reasons why you never participated in an eczema clinical trial?** 367 major themes in 281 responses		
Awareness	Doctor never said anything or recommend, never heard of them/none around	162 (57.7)
No interest	Never considered participation, no motivation to participate	70 (24.9)
Eligibility	Not severe enough/new diagnosis, patient age too young	67 (23.8)
Fear/risks/unknowns of trials	Didn't know outcome, fear of worsening, clinical trial safety	43 (15.3)
Accessibility	Didn't have time/money, participation uncomfortable or burdensome	25 (8.9)
**What would motivate you to decide to explore eczema clinical trial opportunities?** 369 major themes in 281 responses		
Burden of disease	Condition not improving/worsening, dislike having AD self/other, disturbing daily life, improve eczema, treat eczema self/other	161 (57.3)
Out of options/out of control	Frustrated/desperation, access otherwise unavailable care, wants other treatment options	79 (28.1)
Accessibility	Clinical trial availability [timing/close by], if trial were easier to participate in, physician recommendation	59 (21.0)
Fear/risk with/unknowns of trials	Clinical trial safety, fear of side effects from experimental treatment, how trial is presented/introduced, pharmaceuticals‐dislike/distrust, potential to not receive treatment/fear of placebo	48 (17.1)
Altruism	Help researchers/others, contribute to research	22 (7.8)

*Note*: Responses that did not address the question asked were excluded.

### 3.4 Characteristics of those who considered CTs and/or participated

**FIGURE 1:**
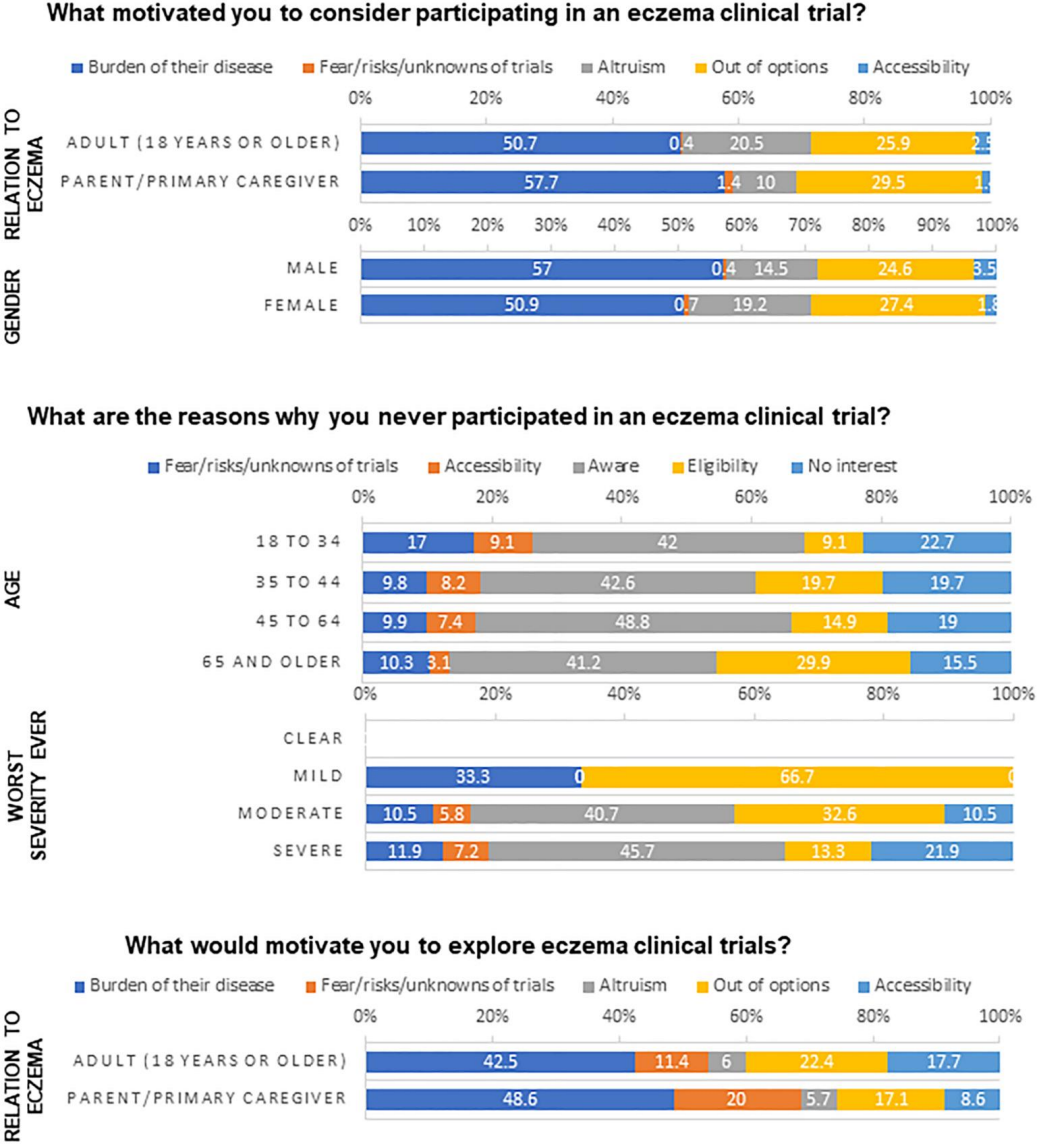
Respondent characteristics that had significantly different distributions of major themes.

We examined the association between respondent demographics and their views on barriers and facilitators of CTP (Significant associations in Figure 1; full analyses in Tables S1–S8 and Figures [Link], [Link], [Link], [Link]).

Among already‐interested respondents, in response to what motivated them to consider CTP, we found a significant association between motivation theme distribution and gender (*p* = 0.008). However, we were not able to specify which motivation themes were significantly different since a post‐hoc analysis yielded no significant result. We observed trends towards self‐identified men identifying disease burden as a motivator and self‐identified women having altruistic motivation to consider participating. We also observed a significant association between motivation theme distribution and whether the respondent was a patient or caregiver (*p* = 0.002). A post‐hoc analysis revealed caregivers had significantly fewer responses coded for altruism compared to patients (*p* = 0.001). We did not observe any significant differences in motivation theme distribution among the other characteristics categories.

We observed no significant association between patient demographics and barriers to enrolling in CTs among those who attempted or had participated in the past. Small sample size for responses to issues getting in CTs prevented our interpretation of those results.

### 3.5 Describing issues completing CTs once enroled

Among the subgroup of participants who previously participated in eczema clinical trials, we observed no significant association between patient demographics and barriers to remaining in CTs. Participants in this subgroup were asked of their difficulties remaining in a CT. Responses centred along the burden of trial participation such as washout periods and difficulties fulfiling trial requirements, and negative participant experiences such as inappropriate investigator conduct and treatments' side effects and ineffectiveness.

### 3.6 Characteristics of those who never considered CTs

When exploring responses to why an individual or their child had never participated in a CT, we observed a significant association with age (*p* = 0.024). Post hoc analyses showed significant differences with the eligibility theme distribution, with the least mentions coming from the 18–34 age group and the most mentions from the 65 and older age group (*p* = 0.012). We also observed a significant association with self‐reported eczema severity when at its worst (*p* = 0.002), with a post‐hoc analysis showing a significant difference with the eligibility distribution (*p* < 0.001). Those who were mild when at their worst cited eligibility twice as much as those who were moderate and even more still than those who were severe. We did not observe any significant associations with theme distribution among the other characteristics categories.

When asked what would motivate this group to explore CTs in the future, we observed a significant association between response theme distribution and whether the respondent was a patient or caregiver (*p* = 0.034), while a post‐hoc analysis revealed no significant difference for any specific theme, we observed a trend towards patients' concern of trial accessibility and caregivers' fears/risks/unknowns of trials. We did not find any other significant associations in response distributions for this question.

### 3.7 Characterising the subgroup of participants who previously never considered and now most likely to consider CTP

We further explored an important subgroup—respondents who had not yet considered CTP but were “extremely likely” to consider participating—by conducting a secondary analysis to identify what was preventing them from participating (See Tables S5 and S6). This subgroup accounted for 19.8% (55) of the respondent population who never considered CTP. We found that the current severity level for any eczema diagnosis (*p* = 0.041) and the current severity level for AD (*p* = 0.050) were significantly associated with this subgroup; in other words, these respondents who hadn't participated yet but were extremely likely to consider had worse eczema at the time of taking the survey than the other survey participants. We found no significant differences in open‐ended responses to reasons why they/their child never participated and what would motivate them to explore CTP.

### 3.8 Characterising the subgroup of participants who previously never considered and now least likely to consider CTP

We also investigated the subgroup of respondents who would be “hardest to reach”—respondents who had not yet considered CTP and were “extremely unlikely” to consider participating (See Tables S7 and S8). This subgroup accounted for 12.1% (34) of the respondent population who never considered CTP. We found no significant differences in terms of respondent characteristics, but this subgroup did have significantly different distributions of themes for reasons why they never participated (*p* = 0.033). This subgroup cited more fears/risks/unknowns of trials and accessibility barriers, lower awareness and eligibility barriers, and no interest themes.

## 4 DISCUSSION

Our survey results reveal a complex landscape of personal and structural motivators and barriers to CTP, with burden of disease emerging as the most common motivator and awareness as the most common barrier. Older and milder patients had a perception of not being eligible to participate in eczema clinical trials, preventing them from even considering CTP. Caregivers were less motivated to consider CTP by altruism than adult patients, and men and women were more motivated to consider CTP by disease burden and altruism respectively. Among those who never considered CTP before, those who were most likely to participate had more severe eczema and more severe AD while those who were least likely to participate cited more fears/risks/unknowns of trials and accessibility barriers. We did not observe any differences based on race/ethnicity and urban/rural status. Several response themes statistically varied by age, gender, and respondent relationship to eczema (caregiver vs. patient).

### 4.1 Discussion of those who considered CTs and/or participated

Patients and caregivers who had considered or actually participated in eczema CTs were motivated mainly by high burden of their disease, the perception of being out of options, and altruism. Recognising this can benefit CTP, it is also imperative not to advertise CTs as an alternative therapeutic route (therapeutic misconception).^20^ With the emergence of many new therapeutic treatments, setting up realistic expectations and remaining honest about the possibility of treatment failure or placebo is important to convey.

Our finding of the importance of altruism is consistent with similar work. A single institution survey of patients and parents of children with AD reported similar results: their top three motivators for adult patients who had participated in research were that other patients with eczema may benefit, contributing to the advancement of knowledge of the field, and their feeling that the study's objectives were important.^14^ Parents of children with AD had the same top factors motivating their decision to participation, but the order changed so that feeling that the study's objective were important was first—a motivator that could be considered the least altruistic and perhaps leading to most direct benefit for the child participant.^14^ In our study, we found a similar significant difference among those who considered or actually participated, where caregivers described altruistic motives less than adult patients. These data contrast with results in other fields such as cancer in which altruism has been reported to either not differ between adult patients and caregivers or not have a significant effect on CTP.^21^, ^22^

Eligibility and accessibility barriers were the most cited reasons for issues getting into CTs. Most response codes referenced age and severity barriers to CT enrolment. Further, eczema is a highly comorbid disease. Elevated age and eczema‐related comorbidities may necessitate CT‐exclusionary treatments, and many responses citied age and eczema‐related comorbidities and their respective treatments as barriers to CTP, .consistent with studies identifying restrictive eligibility criteria as being major barriers to CTP.^23^, ^24^ Researchers looking to improve participation could aim to design CTs that allow for more inclusive eligibility criteria to encourage participation from a diverse set of patients. Adopting less restrictive eligibility criteria may also improve study generalisability and increase the speed of study treatment approval.

Accessibility was a prominent barrier to CT enrolment and included response codes such as “compensation/money,” “trial participation too burdensome,” or “scheduling conflicts,” indicating not only personal but structural barriers. This is a well‐understood barrier. Davis et al., reported significantly higher rates of CTP and awareness in adults living within 100 miles of Clinical and Translational Science Award sites.^25^ Poor trial accessibility and disruptions of work and other daily life activities contribute to a larger barrier of financial burdens associated with trial participation, which many studies have also reported as a deterrent to trial participation.^12^, ^26^, ^27^ Improving accessibility, such as reducing amount of trial visits, time commitment, cost of travel, location, and invasive procedures may help address retention barriers as well. Though the total amount of responses were low, most (64.7%) responses to difficulties remaining in a trial were due to trial burden and accessibility issues, which matches other published studies on CT retention challenges.^28^ Advances in teledermatology and decentralised CT models, which transfer many study activities from study sites to local clinics and participant homes, can help to overcome this barrier.^29^

### 4.2 Discussion of those who never considered CTs

Patients and caregivers who had never considered eczema CTPmainly cited awareness, followed by reiterating their non‐interest in CTP in response to “what are the reasons why you never participated in an eczema CT”. This aligns with the data found by Chu et al., who identified CT awareness as a significant predictor of willingness for participation.^30^ AD patients and parents of children with AD surveyed by Patel and Silverberg had rarely (10.2% and 9.4% respectively) been offered CTP opportunities, though of those who were, high percentages (72.4% and 69.2%, respectively) agreed to participate, indicating high value for addressing this barrier.^14^ Healthcare providers have great potential to raise awareness of CTs in general as well as refer patients to CTs where appropriate. Practicing shared decision making in the clinical setting can greatly facilitate this by letting the patient know CTs are an option, helping the healthcare provider learn if the patient is interested, and discussing any potential concerns that exist. Awareness codes encompassed responses that described general awareness, presence of nearby trials, and physician recommendations.

Perceived eligibility was the third most cited theme for why respondents never considered CTP and more theme volume was significantly associated with increased age and decreased worst eczema severity ever. Responses described feeling like they are not severe enough, dealing with a new diagnosis, or that their age made them ineligible. Similar to awareness for this group, it may be a matter of educating patients on the opportunities for eczema CTs, with emphasis specific to older and milder patients, which disproportionally cited eligibility concerns. Taken together, these data suggest that improved messaging through physicians, patient advocacy groups, and industry partners as well as physician engagement could lead to improved participant recruitment.

### 4.3 Discussion of subgroups among those who never considered CTs

Finally, there was a subgroup of eczema patients and caregivers who never considered CTP and who were extremely likely to consider. While our analysis did not identify anything unique about their reported motivations or barriers, this subgroup had significantly different severity based on their worst eczema and severity among those diagnosed with AD, confirming an important health characteristic that identifies interested candidates.

Fear/risks/unknowns of CTP was not a prominent theme, comprising only 15.3% of reasons why a respondent never participated and 17.1% of reasons a respondent would explore a trial in the future if this barrier were addressed. This demonstrates a duality of concerns, where the presence of one is a barrier and the removal acts as a motivator. However, among those who never considered CTP and were extremely unlikely to, we observed more responses that mentioned fears/risks/unknowns of trials when describing reasons why they never participated. Our data aligns with other published studies on CTP and suggests that fear of risks/unknowns in CTs, though infrequent, is a highly potent barrier, but one that could be mitigated if healthcare providers address fears or risks patients may have regarding enrolment and participation.^14^

### 4.4 Strengths and limitations

Strengths of this study include large sample size and diverse patient and caregiver population of varying eczema types. This allows for a more inclusive and holistic view of CT experiences. The addition of patient/caregiver‐reported data also allows for a unique perspective on CT experience. Limitations of this study include potential recall bias, as respondents were asked to recall their motivations and barriers to CTP of any time frame, and selection bias from NEA community members. The limited number of respondents who indicated they had issues getting in and/or staying in made it challenging to draw conclusions about their barriers at that stage of CTP.

### 4.5 Final recommendations and conclusions

The potential role for investigators and healthcare providers to facilitate CT awareness and participation is vast, with far‐reaching potential. Investigators should appreciate their potential attraction of investigational treatments to patients with eczema and caregivers who are not controlled by their existing therapy. However, it is important to establish realistic expectations and purposes of CTs with potential participants. Randomisation and placebo controls are often necessary in clinical research, but the potential to receive a placebo and leaving treatment in the hands of chance is a disincentive to many patients, hindering enrolment.^23^, ^31^ Additionally, distrust in medical research is a well‐established barrier to CTP, with long‐lasting deleterious effect as seen by the effects of the Tuskegee study on African American participation.^32^ Building trust is an important approach to influencing CTP; trust in the CT doctor(s)/site has been identified as an important factor in eczema CTP decision‐making in previous work conducted by Johnson et al. examining data from this survey.^33^ Therefore, it is important for investigators to spend the time educating potential participants about CTs and their broader goals in order to avoid misunderstanding of their investigational nature. This can serve additional purposes: evidence from our group suggests that confidence in the ability to acquire information on available CTs and understanding of CT‐related terms increase CTP interest.^15^ In conclusion, this study adds to our understanding of the complex motivators and barriers to CTP among adult patients and caregivers of paediatric patients with eczema. This exploratory work can inform future direction with research in this area and have practical applications. Healthcare providers have an important role to facilitate shared decision making beyond decisions about treatments to address any fears, concerns, or other barriers patients may have regarding enrolment and participation in CTs. To maximise participation, investigators should tailor their recruitment approach and study design where possible to address the identified motivators and barriers, also establishing clear aims and potential outcomes. Additionally, implementing CT coordinators as patient educators and providing additional online resources are methods that may provide reassurance for patients throughout the course of the trial.

## CONFLICT OF INTEREST STATEMENT

MEJ has no conflicts to disclose; IJT is a salaried employee of the National Eczema Association, WB has no conflicts to disclose, EL has no conflicts to disclose, AK has no conflicts to disclose, ARL is a salaried employee of the National Eczema Association, ELS reports personal fees from AbbVie, Amgen, Arena Pharmaceuticals, Aslan Pharma, Boston Consulting Group, Collective Acumen, LLC (CA), Dermira, Eli Lilly, Evidera, ExcerptaMedica, Forte Bio RX, Galderma, GlaxoSmithKline, Incyte, Janssen, Kyowa Kirin Pharmaceutical Development, Leo Pharm, Medscape LLC, Merck, Pfizer, Physicians World LLC, Regeneron, Roivant, Sanofi‐Genzyme, Trevi therapeutics, Valeant, WebMD. These potential conflicts of interest have been reviewed and managed by OHSU, WSB is a salaried employee of the National Eczema Association, has received grants from Pfizer, and advisory board honoraria from Pfizer and Amgen.

## AUTHOR CONTRIBUTIONS

**Michael Evan Jacobson**: Data curation (Supporting); Formal analysis (Supporting); Visualization (Supporting); Writing – original draft (Equal); Writing – review & editing (Equal). **Isabelle J. Thibau**: Conceptualization (Equal); Data curation (Equal); Formal analysis (Supporting); Investigation (Lead); Methodology (Equal); Project administration (Lead); Resources (Lead); Visualization (Equal); Writing – original draft (Equal); Writing – review & editing (Equal). **Wenelia Baghoomian**: Data curation (Supporting); Formal analysis (Supporting); Writing – original draft (Equal); Writing – review & editing (Supporting). **Emile Latour**: Data curation (Equal); Formal analysis (Equal); Methodology (Equal); Resources (Supporting); Software (Lead); Visualization (Equal); Writing – original draft (Supporting); Writing – review & editing (Supporting). **Ajai Kastala**: Formal analysis (Supporting). **Allison R. Loiselle**: Formal analysis (Supporting); Writing – review & editing (Supporting). **Eric Lawrence Simpson**: Supervision (Supporting); Writing – review & editing (Supporting). **Wendy Smith Begolka**: Conceptualization (Equal); Funding acquisition (Lead); Investigation (Supporting); Project administration (Supporting); Resources (Supporting); Supervision (Lead); Writing – review & editing (Supporting).

## ETHICS STATEMENT

This study's protocol was submitted to Western IRB Copernicus Group and was determined exempt from full review.

## Supporting information

Supporting Information S1

Supporting Information S2

Supporting Information S3

Supporting Information S4

Supporting Information S5

## Data Availability

Research data are not shared.
