# Supplementary material for: Patient and caregiver motivators and barriers to eczema clinical trial participation: Analysis of survey data
Source: Skin Health Dis. 2023 Jun 22;4(5):e259. doi: 10.1002/ski2.259 (PMC11442076; doi:10.1002/ski2.259)
Supplement: Supplementary file 1 — Supporting Information S1 [file SKI2-4-e259-s001.docx]

**Supporting Information**

**Table S1. Among those who actually participated or considered CTP with/without attempt, association of respondent demographics and major themes for what motivated them to participate.**

| **Characteristic** |  | **Number available** | **Overall** | **Burden of their disease** | **Fear/risks/unknowns of trials** | **Altruism** | **Out of options** | **Accessibility** | **p-value** |
| --- | --- | --- | --- | --- | --- | --- | --- | --- | --- |
| n |  |  | 987 | 516 | 6 | 179 | 264 | 22 |  |
| Age, categorical (%) |  | 987 |  |  |  |  |  |  | 0.139 |
|  | 18 to 34 |  | 280 (28.4) | 142 (50.7) | 3 (1.1) | 53 (18.9) | 70 (25.0) | 12 (4.3) |  |
|  | 35 to 44 |  | 230 (23.3) | 116 (50.4) | 2 (0.9) | 37 (16.1) | 71 (30.9) | 4 (1.7) |  |
|  | 45 to 64 |  | 333 (33.7) | 179 (53.8) | 1 (0.3) | 65 (19.5) | 86 (25.8) | 2 (0.6) |  |
|  | 65 and older |  | 144 (14.6) | 79 (54.9) | 0 (0.0) | 24 (16.7) | 37 (25.7) | 4 (2.8) |  |
| Primary connection to eczema (%) |  | 987 |  |  |  |  |  |  | < 0.001 |
|  | Adult (18 years or older) |  | 767 (77.7) | 389 (50.7) | 3 (0.4) | 157 (20.5) | 199 (25.9) | 19 (2.5) |  |
|  | Parent/primary caregiver |  | 220 (22.3) | 127 (57.7) | 3 (1.4) | 22 (10.0) | 65 (29.5) | 3 (1.4) |  |
| Gender (%) |  | 987 |  |  |  |  |  |  | 0.008 |
|  | Male |  | 228 (23.1) | 130 (57.0) | 1 (0.4) | 33 (14.5) | 56 (24.6) | 8 (3.5) |  |
|  | Female |  | 759 (76.9) | 386 (50.9) | 5 (0.7) | 146 (19.2) | 208 (27.4) | 14 (1.8) |  |
| Race x Ethnicity (%) |  | 987 |  |  |  |  |  |  | 0.920 |
|  | Hispanic or Latino |  | 118 (12.0) | 61 (51.7) | 1 (0.8) | 23 (19.5) | 31 (26.3) | 2 (1.7) |  |
|  | Not Hispanic or Latino, White |  | 631 (63.9) | 324 (51.3) | 3 (0.5) | 120 (19.0) | 170 (26.9) | 14 (2.2) |  |
|  | Not Hispanic or Latino, Black |  | 96 (9.7) | 55 (57.3) | 1 (1.0) | 15 (15.6) | 22 (22.9) | 3 (3.1) |  |
|  | Not Hispanic or Latino, Asian |  | 85 (8.6) | 44 (51.8) | 1 (1.2) | 12 (14.1) | 27 (31.8) | 1 (1.2) |  |
|  | Not Hispanic or Latino, Other |  | 57 (5.8) | 32 (56.1) | 0 (0.0) | 9 (15.8) | 14 (24.6) | 2 (3.5) |  |
| RUCA (%) |  | 987 |  |  |  |  |  |  | 0.982 |
|  | Urban (1 to 3) |  | 884 (89.6) | 463 (52.4) | 5 (0.6) | 160 (18.1) | 236 (26.7) | 20 (2.3) |  |
|  | Rural (4 to 10) |  | 103 (10.4) | 53 (51.5) | 1 (1.0) | 19 (18.4) | 28 (27.2) | 2 (1.9) |  |
| Worst Severity ever (all disease types) (%) |  | 987 |  |  |  |  |  |  | 0.077 |
|  | Clear |  | 0 (0.0) | 0 (NA) | 0 (NA) | 0 (NA) | 0 (NA) | 0 (NA) |  |
|  | Mild |  | 5 (0.5) | 2 (40.0) | 0 (0.0) | 2 (40.0) | 1 (20.0) | 0 (0.0) |  |
|  | Moderate |  | 162 (16.4) | 83 (51.2) | 0 (0.0) | 28 (17.3) | 50 (30.9) | 1 (0.6) |  |
|  | Severe |  | 820 (83.1) | 431 (52.6) | 6 (0.7) | 149 (18.2) | 213 (26.0) | 21 (2.6) |  |

**Table S2. Among those who actually participated or considered CTP with/without attempt, association of respondent demographics and major themes for why they had issues getting in.**

| **Characteristic** |  | **Number available** | **Overall** | **Eligibility** | **Fear/risks/unknowns of trials** | **Burden/accessibility** | **Administrative** | **Other** | **p-value** |
| --- | --- | --- | --- | --- | --- | --- | --- | --- | --- |
| n |  |  | 169 | 67 | 6 | 54 | 27 | 15 |  |
| Age, categorical (%) |  | 169 |  |  |  |  |  |  | 0.941 |
|  | 18 to 34 |  | 43 (25.4) | 17 (39.5) | 2 (4.7) | 15 (34.9) | 6 (14.0) | 3 (7.0) |  |
|  | 35 to 44 |  | 37 (21.9) | 16 (43.2) | 2 (5.4) | 9 (24.3) | 6 (16.2) | 4 (10.8) |  |
|  | 45 to 64 |  | 67 (39.6) | 24 (35.8) | 2 (3.0) | 23 (34.3) | 13 (19.4) | 5 (7.5) |  |
|  | 65 and older |  | 22 (13.0) | 10 (45.5) | 0 (0.0) | 7 (31.8) | 2 (9.1) | 3 (13.6) |  |
| Primary connection to eczema (%) |  | 169 |  |  |  |  |  |  | 0.468 |
|  | Adult (18 years or older) |  | 124 (73.4) | 49 (39.5) | 3 (2.4) | 42 (33.9) | 18 (14.5) | 12 (9.7) |  |
|  | Parent/primary caregiver |  | 45 (26.6) | 18 (40.0) | 3 (6.7) | 12 (26.7) | 9 (20.0) | 3 (6.7) |  |
| Gender (%) |  | 169 |  |  |  |  |  |  | 0.710 |
|  | Male |  | 43 (25.4) | 15 (34.9) | 1 (2.3) | 13 (30.2) | 9 (20.9) | 5 (11.6) |  |
|  | Female |  | 126 (74.6) | 52 (41.3) | 5 (4.0) | 41 (32.5) | 18 (14.3) | 10 (7.9) |  |
| Race x Ethnicity (%) |  | 169 |  |  |  |  |  |  | 0.546 |
|  | Hispanic or Latino |  | 8 (4.7) | 3 (37.5) | 1 (12.5) | 1 (12.5) | 3 (37.5) | 0 (0.0) |  |
|  | Not Hispanic or Latino, White |  | 115 (68.0) | 47 (40.9) | 2 (1.7) | 36 (31.3) | 18 (15.7) | 12 (10.4) |  |
|  | Not Hispanic or Latino, Black |  | 12 (7.1) | 6 (50.0) | 0 (0.0) | 5 (41.7) | 1 (8.3) | 0 (0.0) |  |
|  | Not Hispanic or Latino, Asian |  | 20 (11.8) | 5 (25.0) | 2 (10.0) | 8 (40.0) | 4 (20.0) | 1 (5.0) |  |
|  | Not Hispanic or Latino, Other |  | 14 (8.3) | 6 (42.9) | 1 (7.1) | 4 (28.6) | 1 (7.1) | 2 (14.3) |  |
| RUCA (%) |  | 169 |  |  |  |  |  |  | 0.243 |
|  | Urban (1 to 3) |  | 155 (91.7) | 63 (40.6) | 6 (3.9) | 46 (29.7) | 26 (16.8) | 14 (9.0) |  |
|  | Rural (4 to 10) |  | 14 (8.3) | 4 (28.6) | 0 (0.0) | 8 (57.1) | 1 (7.1) | 1 (7.1) |  |
| Worst Severity ever (all disease types) (%) |  | 169 |  |  |  |  |  |  | 0.473 |
|  | Clear |  | 0 (0.0) | 0 (NA) | 0 (NA) | 0 (NA) | 0 (NA) | 0 (NA) |  |
|  | Mild |  | 1 (0.6) | 0 (0.0) | 0 (0.0) | 0 (0.0) | 1 (100.0) | 0 (0.0) |  |
|  | Moderate |  | 23 (13.6) | 12 (52.2) | 0 (0.0) | 5 (21.7) | 3 (13.0) | 3 (13.0) |  |
|  | Severe |  | 145 (85.8) | 55 (37.9) | 6 (4.1) | 49 (33.8) | 23 (15.2) | 12 (8.3) |  |

**Table S3. Among those who never considered CTP, association of respondent demographics and major themes for why they never participated.**

| **Characteristic** |  | **Number Available** | **Overall** | **Fear/risks/unknowns of trials** | **Accessibility** | **Aware** | **Eligibility** | **No interest** | **p-value** |
| --- | --- | --- | --- | --- | --- | --- | --- | --- | --- |
| n |  |  | 367 | 43 | 25 | 162 | 67 | 70 |  |
| Age, categorical (%) |  | 367 |  |  |  |  |  |  | 0.024 |
|  | 18 to 34 |  | 88 (24.0) | 15 (17.0) | 8 (9.1) | 37 (42.0) | 8 (9.1) | 20 (22.7) |  |
|  | 35 to 44 |  | 61 (16.6) | 6 (9.8) | 5 (8.2) | 26 (42.6) | 12 (19.7) | 12 (19.7) |  |
|  | 45 to 64 |  | 121 (33.0) | 12 (9.9) | 9 (7.4) | 59 (48.8) | 18 (14.9) | 23 (19.0) |  |
|  | 65 and older |  | 97 (26.4) | 10 (10.3) | 3 (3.1) | 40 (41.2) | 29 (29.9) | 15 (15.5) |  |
| Primary connection to eczema (%) |  | 367 |  |  |  |  |  |  | 0.992 |
|  | Adult (18 years or older) |  | 296 (80.7) | 34 (11.5) | 20 (6.8) | 131 (44.3) | 54 (18.2) | 57 (19.3) |  |
|  | Parent/primary caregiver |  | 71 (19.3) | 9 (12.7) | 5 (7.0) | 31 (43.7) | 13 (18.3) | 13 (18.3) |  |
| Gender (%) |  | 367 |  |  |  |  |  |  | 0.133 |
|  | Male |  | 100 (27.2) | 7 (7.0) | 9 (9.0) | 47 (47.0) | 22 (22.0) | 15 (15.0) |  |
|  | Female |  | 267 (72.8) | 36 (13.5) | 16 (6.0) | 115 (43.1) | 45 (16.9) | 55 (20.6) |  |
| Race x Ethnicity (%) |  | 367 |  |  |  |  |  |  | 0.537 |
|  | Hispanic or Latino |  | 30 (8.2) | 3 (10.0) | 2 (6.7) | 14 (46.7) | 5 (16.7) | 6 (20.0) |  |
|  | Not Hispanic or Latino, White |  | 266 (72.5) | 31 (11.7) | 17 (6.4) | 110 (41.4) | 55 (20.7) | 53 (19.9) |  |
|  | Not Hispanic or Latino, Black |  | 20 (5.4) | 1 (5.0) | 1 (5.0) | 13 (65.0) | 2 (10.0) | 3 (15.0) |  |
|  | Not Hispanic or Latino, Asian |  | 33 (9.0) | 7 (21.2) | 3 (9.1) | 15 (45.5) | 2 (6.1) | 6 (18.2) |  |
|  | Not Hispanic or Latino, Other |  | 18 (4.9) | 1 (5.6) | 2 (11.1) | 10 (55.6) | 3 (16.7) | 2 (11.1) |  |
| RUCA (%) |  | 367 |  |  |  |  |  |  | 0.188 |
|  | Urban (1 to 3) |  | 322 (87.7) | 39 (12.1) | 19 (5.9) | 141 (43.8) | 63 (19.6) | 60 (18.6) |  |
|  | Rural (4 to 10) |  | 45 (12.3) | 4 (8.9) | 6 (13.3) | 21 (46.7) | 4 (8.9) | 10 (22.2) |  |
| Worst Severity ever (all disease types) (%) |  | 367 |  |  |  |  |  |  | 0.002 |
|  | Clear |  | 0 (0.0) | 0 (NA) | 0 (NA) | 0 (NA) | 0 (NA) | 0 (NA) |  |
|  | Mild |  | 3(0.8) | 1 (33.3) | 0 (0.0) | 0 (0.0) | 2 (66.7) | 0 (0.0) |  |
|  | Moderate |  | 86 (23.4) | 9 (10.5) | 5 (5.8) | 35 (40.7) | 28 (32.6) | 9 (10.5) |  |
|  | Severe |  | 278 (75.7) | 33 (11.9) | 20 (7.2) | 127 (45.7) | 37 (13.3) | 61 (21.9) |  |

**Table S4. Among those who never considered CTP, association of respondent demographics and major themes for what would motivate them to explore CTP.**

| **Characteristic** |  | **Number avaiable** | **Overall** | **Burden of their disease** | **Fear/risks/unknowns of trials** | **Altruism** | **Out of options** | **Accessibility** | **p-value** |
| --- | --- | --- | --- | --- | --- | --- | --- | --- | --- |
| n |  |  | 369 | 161 | 48 | 22 | 79 | 59 |  |
| Age, categorical (%) |  | 369 |  |  |  |  |  |  | 0.060 |
|  | 18 to 34 |  | 90 (24.4) | 38 (42.2) | 11 (12.2) | 10 (11.1) | 13 (14.4) | 18 (20.0) |  |
|  | 35 to 44 |  | 55 (14.9) | 26 (47.3) | 11 (20.0) | 1 (1.8) | 11 (20.0) | 6 (10.9) |  |
|  | 45 to 64 |  | 129 (35.0) | 58 (45.0) | 15 (11.6) | 2 (1.6) | 32 (24.8) | 22 (17.1) |  |
|  | 65 and older |  | 95 (25.7) | 39 (41.1) | 11 (11.6) | 9 (9.5) | 23 (24.2) | 13 (13.7) |  |
| Primary connection to eczema (%) |  | 369 |  |  |  |  |  |  | 0.034 |
|  | Adult (18 years or older) |  | 299 (81.0) | 127 (42.5) | 34 (11.4) | 18 (6.0) | 67 (22.4) | 53 (17.7) |  |
|  | Parent/primary caregiver |  | 70 (19.0) | 34 (48.6) | 14 (20.0) | 4 (5.7) | 12 (17.1) | 6 (8.6) |  |
| Gender (%) |  | 369 |  |  |  |  |  |  | 0.179 |
|  | Male |  | 103 (27.9) | 45 (43.7) | 8 (7.8) | 10 (9.7) | 22 (21.4) | 18 (17.5) |  |
|  | Female |  | 266 (72.1) | 116 (43.6) | 40 (15.0) | 12 (4.5) | 57 (21.4) | 41 (15.4) |  |
| Race x Ethnicity (%) |  | 369 |  |  |  |  |  |  | 0.708 |
|  | Hispanic or Latino |  | 30 (8.1) | 13 (43.3) | 5 (16.7) | 1 (3.3) | 7 (23.3) | 4 (13.3) |  |
|  | Not Hispanic or Latino, White |  | 274 (74.3) | 120 (43.8) | 33 (12.0) | 19 (6.9) | 63 (23.0) | 39 (14.2) |  |
|  | Not Hispanic or Latino, Black |  | 19 (5.1) | 7 (36.8) | 3 (15.8) | 0 (0.0) | 3 (15.8) | 6 (31.6) |  |
|  | Not Hispanic or Latino, Asian |  | 28 (7.6) | 13 (46.4) | 5 (17.9) | 1 (3.6) | 3 (10.7) | 6 (21.4) |  |
|  | Not Hispanic or Latino, Other |  | 18 (4.9) | 8 (44.4) | 2 (11.1) | 1 (5.6) | 3 (16.7) | 4 (22.2) |  |
| RUCA (%) |  | 369 |  |  |  |  |  |  | 0.727 |
|  | Urban (1 to 3) |  | 317 (85.9) | 138 (43.5) | 43 (13.6) | 20 (6.3) | 67 (21.1) | 49 (15.5) |  |
|  | Rural (4 to 10) |  | 52 (14.1) | 23 (44.2) | 5 (9.6) | 2 (3.8) | 12 (23.1) | 10 (19.2) |  |
| Worst Severity ever (all disease types) (%) |  | 369 |  |  |  |  |  |  | 0.389 |
|  | Clear |  | 0 (0.0) | 0 (NA) | 0 (NA) | 0 (NA) | 0 (NA) | 0 (NA) |  |
|  | Mild |  | 5 (1.4) | 3 (60.0) | 1 (20.0) | 0 (0.0) | 1 (20.0) | 0 (0.0) |  |
|  | Moderate |  | 102 (27.6) | 42 (41.2) | 15 (14.7) | 4 (3.9) | 24 (23.5) | 17 (16.7) |  |
|  | Severe |  | 262 (71.0) | 116 (44.3) | 32 (12.2) | 18 (6.9) | 54 (20.6) | 42 (16.0) |  |

**Table S5. Among those who never considered CTP, characteristics of the best untapped subgroup.**

|  |  |  |  |  |  |  |
| --- | --- | --- | --- | --- | --- | --- |
| **Category** | **level** | **Number not missing** | **Overall** | **In Best Untapped** | **Not in Best Untapped** | **p** |
| n |  |  | 281 | 55 | 223 |  |
| Age, continuous (mean (SD)) |  | 281 | 51.22 (17.55) | 50.49 (18.95) | 51.57 (17.17) | 0.684 |
| Age, categorical (%) |  | 281 |  |  |  | 0.339 |
|  | 18 to 34 |  | 68 (24.2) | 17 (30.9) | 49 (22.0) |  |
|  | 35 to 44 |  | 40 (14.2) | 5 (9.1) | 35 (15.7) |  |
|  | 45 to 64 |  | 94 (33.5) | 16 (29.1) | 77 (34.5) |  |
|  | 65 and older |  | 79 (28.1) | 17 (30.9) | 62 (27.8) |  |
| Primary connection to eczema (%) |  | 281 |  |  |  | 0.873 |
|  | Adult (18 years or older) |  | 230 (81.9) | 44 (80.0) | 183 (82.1) |  |
|  | Parent/primary caregiver |  | 51 (18.1) | 11 (20.0) | 40 (17.9) |  |
| Gender (%) |  | 281 |  |  |  | NaN |
|  | Male |  | 84 (29.9) | 20 (36.4) | 62 (27.8) |  |
|  | Female |  | 197 (70.1) | 35 (63.6) | 161 (72.2) |  |
|  | Non-binary |  | 0 (0.0) | 0 (0.0) | 0 (0.0) |  |
|  | Other |  | 0 (0.0) | 0 (0.0) | 0 (0.0) |  |
| Race (%) |  | 277 |  |  |  | NaN |
|  | White |  | 214 (77.3) | 41 (77.4) | 172 (77.8) |  |
|  | Black or African American |  | 21 (7.6) | 3 (5.7) | 17 (7.7) |  |
|  | American Indian or Alaskan Native |  | 1 (0.4) | 1 (1.9) | 0 (0.0) |  |
|  | Native Hawaiian or Pacific Islander |  | 0 (0.0) | 0 (0.0) | 0 (0.0) |  |
|  | Asian or Asian American |  | 26 (9.4) | 4 (7.5) | 22 (10.0) |  |
|  | Some other race or ethnicity |  | 9 (3.2) | 2 (3.8) | 6 (2.7) |  |
|  | Multiracial |  | 6 (2.2) | 2 (3.8) | 4 (1.8) |  |
|  | I don’t know/prefer not to answer |  | 0 (0.0) | 0 (0.0) | 0 (0.0) |  |
| Ethnicity (%) |  | 281 |  |  |  | 0.079 |
|  | Hispanic |  | 23 (8.2) | 8 (14.5) | 14 (6.3) |  |
|  | Not Hispanic |  | 258 (91.8) | 47 (85.5) | 209 (93.7) |  |
| Race x Ethnicity (%) |  | 281 |  |  |  | 0.073 |
|  | Hispanic or Latino |  | 23 (8.2) | 8 (14.5) | 14 (6.3) |  |
|  | Not Hispanic or Latino, White |  | 202 (71.9) | 37 (67.3) | 164 (73.5) |  |
|  | Not Hispanic or Latino, Black |  | 19 (6.8) | 2 (3.6) | 16 (7.2) |  |
|  | Not Hispanic or Latino, Asian |  | 24 (8.5) | 3 (5.5) | 21 (9.4) |  |
|  | Not Hispanic or Latino, Other |  | 13 (4.6) | 5 (9.1) | 8 (3.6) |  |
| RUCA (%) |  | 281 |  |  |  | 0.866 |
|  | Urban (1 to 3) |  | 245 (87.2) | 47 (85.5) | 195 (87.4) |  |
|  | Rural (4 to 10) |  | 36 (12.8) | 8 (14.5) | 28 (12.6) |  |
| Diagnosed with by a healthcare provider with Atopic Dermatitis (%) |  | 281 |  |  |  | 0.424 |
|  | 0 |  | 59 (21.0) | 9 (16.4) | 50 (22.4) |  |
|  | Diagnosed with AD |  | 222 (79.0) | 46 (83.6) | 173 (77.6) |  |
| Worst severity: Atopic Dermatitis (%) |  | 222 |  |  |  | NaN |
|  | Clear |  | 0 (0.0) | 0 (0.0) | 0 (0.0) |  |
|  | Mild |  | 3 (1.4) | 0 (0.0) | 3 (1.7) |  |
|  | Moderate |  | 62 (27.9) | 12 (26.1) | 50 (28.9) |  |
|  | Severe |  | 157 (70.7) | 34 (73.9) | 120 (69.4) |  |
| Worst severity: Contact Dermatitis (%) |  | 87 |  |  |  | 0.803 |
|  | Clear |  | 1 (1.1) | 0 (0.0) | 1 (1.5) |  |
|  | Mild |  | 5 (5.7) | 1 (4.5) | 4 (6.2) |  |
|  | Moderate |  | 33 (37.9) | 10 (45.5) | 23 (35.4) |  |
|  | Severe |  | 48 (55.2) | 11 (50.0) | 37 (56.9) |  |
| Worst severity: Dyshidrotic Eczema (%) |  | 30 |  |  |  | NaN |
|  | Clear |  | 0 (0.0) | 0 (0.0) | 0 (0.0) |  |
|  | Mild |  | 1 (3.3) | 0 (0.0) | 1 (3.8) |  |
|  | Moderate |  | 5 (16.7) | 0 (0.0) | 5 (19.2) |  |
|  | Severe |  | 24 (80.0) | 4 (100.0) | 20 (76.9) |  |
| Worst severity: Hand Eczema (%) |  | 87 |  |  |  | NaN |
|  | Clear |  | 0 (0.0) | 0 (0.0) | 0 (0.0) |  |
|  | Mild |  | 3 (3.4) | 1 (4.8) | 2 (3.0) |  |
|  | Moderate |  | 20 (23.0) | 7 (33.3) | 13 (19.7) |  |
|  | Severe |  | 64 (73.6) | 13 (61.9) | 51 (77.3) |  |
| Worst severity: Neurodermatitis (%) |  | 7 |  |  |  | NaN |
|  | Clear |  | 0 (0.0) | 0 (0.0) | 0 (0.0) |  |
|  | Mild |  | 0 (0.0) | 0 (0.0) | 0 (0.0) |  |
|  | Moderate |  | 2 (28.6) | 1 (33.3) | 1 (33.3) |  |
|  | Severe |  | 5 (71.4) | 2 (66.7) | 2 (66.7) |  |
| Worst severity: Nummular Eczema (%) |  | 25 |  |  |  | NaN |
|  | Clear |  | 0 (0.0) | 0 (0.0) | 0 (0.0) |  |
|  | Mild |  | 4 (16.0) | 0 (0.0) | 4 (19.0) |  |
|  | Moderate |  | 8 (32.0) | 1 (25.0) | 7 (33.3) |  |
|  | Severe |  | 13 (52.0) | 3 (75.0) | 10 (47.6) |  |
| Worst severity: Seborrheic Dermatitis (%) |  | 40 |  |  |  | NaN |
|  | Clear |  | 0 (0.0) | 0 (0.0) | 0 (0.0) |  |
|  | Mild |  | 3 (7.5) | 1 (12.5) | 2 (6.5) |  |
|  | Moderate |  | 16 (40.0) | 5 (62.5) | 10 (32.3) |  |
|  | Severe |  | 21 (52.5) | 2 (25.0) | 19 (61.3) |  |
| Worst severity: Stasis Dermatitis (%) |  | 3 |  |  |  | NaN |
|  | Clear |  | 0 (0.0) | 0 (NaN) | 0 (0.0) |  |
|  | Mild |  | 0 (0.0) | 0 (NaN) | 0 (0.0) |  |
|  | Moderate |  | 1 (33.3) | 0 (NaN) | 1 (33.3) |  |
|  | Severe |  | 2 (66.7) | 0 (NaN) | 2 (66.7) |  |
| Severity now: Atopic Dermatitis (%) |  | 222 |  |  |  | 0.050 |
|  | Clear |  | 13 (5.9) | 2 (4.3) | 11 (6.4) |  |
|  | Mild |  | 73 (32.9) | 9 (19.6) | 63 (36.4) |  |
|  | Moderate |  | 94 (42.3) | 21 (45.7) | 72 (41.6) |  |
|  | Severe |  | 42 (18.9) | 14 (30.4) | 27 (15.6) |  |
| Severity now: Contact Dermatitis (%) |  | 87 |  |  |  | 0.229 |
|  | Clear |  | 14 (16.1) | 2 (9.1) | 12 (18.5) |  |
|  | Mild |  | 31 (35.6) | 10 (45.5) | 21 (32.3) |  |
|  | Moderate |  | 33 (37.9) | 6 (27.3) | 27 (41.5) |  |
|  | Severe |  | 9 (10.3) | 4 (18.2) | 5 (7.7) |  |
| Severity now: Dyshidrotic Eczema (%) |  | 30 |  |  |  | 0.421 |
|  | Clear |  | 3 (10.0) | 0 (0.0) | 3 (11.5) |  |
|  | Mild |  | 13 (43.3) | 3 (75.0) | 10 (38.5) |  |
|  | Moderate |  | 8 (26.7) | 0 (0.0) | 8 (30.8) |  |
|  | Severe |  | 6 (20.0) | 1 (25.0) | 5 (19.2) |  |
| Severity now: Hand Eczema (%) |  | 87 |  |  |  | 0.358 |
|  | Clear |  | 2 (2.3) | 0 (0.0) | 2 (3.0) |  |
|  | Mild |  | 26 (29.9) | 5 (23.8) | 21 (31.8) |  |
|  | Moderate |  | 41 (47.1) | 9 (42.9) | 32 (48.5) |  |
|  | Severe |  | 18 (20.7) | 7 (33.3) | 11 (16.7) |  |
| Severity now: Neurodermatitis (%) |  | 7 |  |  |  | NaN |
|  | Clear |  | 0 (0.0) | 0 (0.0) | 0 (0.0) |  |
|  | Mild |  | 1 (14.3) | 1 (33.3) | 0 (0.0) |  |
|  | Moderate |  | 4 (57.1) | 1 (33.3) | 2 (66.7) |  |
|  | Severe |  | 2 (28.6) | 1 (33.3) | 1 (33.3) |  |
| Severity now: Nummular Eczema (%) |  | 25 |  |  |  | 0.125 |
|  | Clear |  | 5 (20.0) | 0 (0.0) | 5 (23.8) |  |
|  | Mild |  | 13 (52.0) | 1 (25.0) | 12 (57.1) |  |
|  | Moderate |  | 4 (16.0) | 2 (50.0) | 2 (9.5) |  |
|  | Severe |  | 3 (12.0) | 1 (25.0) | 2 (9.5) |  |
| Severity now: Seborrheic Dermatitis (%) |  | 40 |  |  |  | 0.386 |
|  | Clear |  | 7 (17.5) | 1 (12.5) | 6 (19.4) |  |
|  | Mild |  | 15 (37.5) | 5 (62.5) | 10 (32.3) |  |
|  | Moderate |  | 14 (35.0) | 1 (12.5) | 12 (38.7) |  |
|  | Severe |  | 4 (10.0) | 1 (12.5) | 3 (9.7) |  |
| Severity now: Stasis Dermatitis (%) |  | 3 |  |  |  | NaN |
|  | Clear |  | 0 (0.0) | 0 (NaN) | 0 (0.0) |  |
|  | Mild |  | 0 (0.0) | 0 (NaN) | 0 (0.0) |  |
|  | Moderate |  | 2 (66.7) | 0 (NaN) | 2 (66.7) |  |
|  | Severe |  | 1 (33.3) | 0 (NaN) | 1 (33.3) |  |
| Previous CT participation/attempts/consideration (%) |  | 281 |  |  |  | NaN |
|  | 1 |  | 0 (0.0) | 0 (0.0) | 0 (0.0) |  |
|  | 2 |  | 0 (0.0) | 0 (0.0) | 0 (0.0) |  |
|  | 3 or more |  | 0 (0.0) | 0 (0.0) | 0 (0.0) |  |
|  | None, but considered (and tried) |  | 0 (0.0) | 0 (0.0) | 0 (0.0) |  |
|  | None, but considered (never tried) |  | 0 (0.0) | 0 (0.0) | 0 (0.0) |  |
|  | None, never considered |  | 281 (100.0) | 55 (100.0) | 223 (100.0) |  |
| Worst severity ever (all types) (%) |  | 281 |  |  |  | NaN |
|  | Clear |  | 0 (0.0) | 0 (0.0) | 0 (0.0) |  |
|  | Mild |  | 4 (1.4) | 0 (0.0) | 4 (1.8) |  |
|  | Moderate |  | 64 (22.8) | 13 (23.6) | 51 (22.9) |  |
|  | Severe |  | 213 (75.8) | 42 (76.4) | 168 (75.3) |  |
| Worst severity now (all types) (%) |  | 281 |  |  |  | 0.041 |
|  | Clear |  | 13 (4.6) | 1 (1.8) | 12 (5.4) |  |
|  | Mild |  | 74 (26.3) | 11 (20.0) | 63 (28.3) |  |
|  | Moderate |  | 130 (46.3) | 23 (41.8) | 105 (47.1) |  |
|  | Severe |  | 64 (22.8) | 20 (36.4) | 43 (19.3) |  |

**Table S6. Response theme distributions for best untapped subgroup.**

|  |  |  |  |  |  |
| --- | --- | --- | --- | --- | --- |
| **Feature** |  | **Overall** | **In Best Untapped** | **Not in Best Untapped** | **p-value** |
| What are the reasons why you never participated in an eczema clinical trial? |  | 369 | 86 | 280 |  |
|  |  |  |  |  | 0.060 |
|  | Fear/risks/unknowns of trials | 43 (11.7) | 2 (2.8) | 41 (14.0) |  |
|  | Accessibility | 25 (6.8) | 5 (7.0) | 19 (6.5) |  |
|  | Aware | 162 (44.1) | 30 (42.3) | 130 (44.4) |  |
|  | Eligibility | 67 (18.3) | 16 (22.5) | 51 (17.4) |  |
|  | No interest | 70 (19.1) | 18 (25.4) | 52 (17.7) |  |
|  |  |  |  |  |  |
| What would motivate you to explore eczema clinical trials? |  | 369 | 86 | 280 |  |
|  |  |  |  |  | 0.154 |
|  | Burden of their disease | 161 (43.6) | 43 (50.0) | 117 (41.8) |  |
|  | Fear/risks/unknowns of trials | 48 (13.0) | 7 (8.1) | 41 (14.6) |  |
|  | Altruism | 22 (6.0) | 5 (5.8) | 17 (6.1) |  |
|  | Out of options | 79 (21.4) | 20 (23.3) | 58 (20.7) |  |
|  | Accessibility | 59 (16.0) | 11 (12.8) | 47 (16.8) |  |
|  |  |  |  |  |  |

**Table S7. Among those who never considered CTP, demographics of the hardest to reach subgroup.**

|  |  |  |  |  |  |  |
| --- | --- | --- | --- | --- | --- | --- |
| **Category** | **level** | **num_not_miss** | **Overall** | **In Hardest to Reach** | **Not in Hardest to Reach** | **p** |
| n |  |  | 281 | 34 | 244 |  |
| Age, continuous (mean (SD)) |  | 281 | 51.22 (17.55) | 51.85 (15.36) | 51.28 (17.81) | 0.859 |
| Age, categorical (%) |  | 281 |  |  |  | 0.353 |
|  | 18 to 34 |  | 68 (24.2) | 6 (17.6) | 60 (24.6) |  |
|  | 35 to 44 |  | 40 (14.2) | 4 (11.8) | 36 (14.8) |  |
|  | 45 to 64 |  | 94 (33.5) | 16 (47.1) | 77 (31.6) |  |
|  | 65 and older |  | 79 (28.1) | 8 (23.5) | 71 (29.1) |  |
| Primary connection to eczema (%) |  | 281 |  |  |  | 0.195 |
|  | Adult (18 years or older) |  | 230 (81.9) | 31 (91.2) | 196 (80.3) |  |
|  | Parent/primary caregiver |  | 51 (18.1) | 3 (8.8) | 48 (19.7) |  |
| Gender (%) |  | 281 |  |  |  | NaN |
|  | Male |  | 84 (29.9) | 6 (17.6) | 76 (31.1) |  |
|  | Female |  | 197 (70.1) | 28 (82.4) | 168 (68.9) |  |
|  | Non-binary |  | 0 (0.0) | 0 (0.0) | 0 (0.0) |  |
|  | Other |  | 0 (0.0) | 0 (0.0) | 0 (0.0) |  |
| Race (%) |  | 277 |  |  |  | NaN |
|  | White |  | 214 (77.3) | 27 (81.8) | 186 (77.2) |  |
|  | Black or African American |  | 21 (7.6) | 4 (12.1) | 16 (6.6) |  |
|  | American Indian or Alaskan Native |  | 1 (0.4) | 0 (0.0) | 1 (0.4) |  |
|  | Native Hawaiian or Pacific Islander |  | 0 (0.0) | 0 (0.0) | 0 (0.0) |  |
|  | Asian or Asian American |  | 26 (9.4) | 2 (6.1) | 24 (10.0) |  |
|  | Some other race or ethnicity |  | 9 (3.2) | 0 (0.0) | 8 (3.3) |  |
|  | Multiracial |  | 6 (2.2) | 0 (0.0) | 6 (2.5) |  |
|  | I don’t know/prefer not to answer |  | 0 (0.0) | 0 (0.0) | 0 (0.0) |  |
| Ethnicity (%) |  | 281 |  |  |  | 0.897 |
|  | Hispanic |  | 23 (8.2) | 2 (5.9) | 20 (8.2) |  |
|  | Not Hispanic |  | 258 (91.8) | 32 (94.1) | 224 (91.8) |  |
| Race x Ethnicity (%) |  | 281 |  |  |  | 0.390 |
|  | Hispanic or Latino |  | 23 (8.2) | 2 (5.9) | 20 (8.2) |  |
|  | Not Hispanic or Latino, White |  | 202 (71.9) | 26 (76.5) | 175 (71.7) |  |
|  | Not Hispanic or Latino, Black |  | 19 (6.8) | 4 (11.8) | 14 (5.7) |  |
|  | Not Hispanic or Latino, Asian |  | 24 (8.5) | 2 (5.9) | 22 (9.0) |  |
|  | Not Hispanic or Latino, Other |  | 13 (4.6) | 0 (0.0) | 13 (5.3) |  |
| RUCA (%) |  | 281 |  |  |  | 1.000 |
|  | Urban (1 to 3) |  | 245 (87.2) | 30 (88.2) | 212 (86.9) |  |
|  | Rural (4 to 10) |  | 36 (12.8) | 4 (11.8) | 32 (13.1) |  |
| Diagnosed with by a healthcare provider with Atopic Dermatitis (%) |  | 281 |  |  |  | 1.000 |
|  | 0 |  | 59 (21.0) | 7 (20.6) | 52 (21.3) |  |
|  | Diagnosed with AD |  | 222 (79.0) | 27 (79.4) | 192 (78.7) |  |
| Worst severity: Atopic Dermatitis (%) |  | 222 |  |  |  | NaN |
|  | Clear |  | 0 (0.0) | 0 (0.0) | 0 (0.0) |  |
|  | Mild |  | 3 (1.4) | 1 (3.7) | 2 (1.0) |  |
|  | Moderate |  | 62 (27.9) | 7 (25.9) | 55 (28.6) |  |
|  | Severe |  | 157 (70.7) | 19 (70.4) | 135 (70.3) |  |
| Worst severity: Contact Dermatitis (%) |  | 87 |  |  |  | 0.898 |
|  | Clear |  | 1 (1.1) | 0 (0.0) | 1 (1.4) |  |
|  | Mild |  | 5 (5.7) | 1 (7.7) | 4 (5.4) |  |
|  | Moderate |  | 33 (37.9) | 4 (30.8) | 29 (39.2) |  |
|  | Severe |  | 48 (55.2) | 8 (61.5) | 40 (54.1) |  |
| Worst severity: Dyshidrotic Eczema (%) |  | 30 |  |  |  | NaN |
|  | Clear |  | 0 (0.0) | 0 (0.0) | 0 (0.0) |  |
|  | Mild |  | 1 (3.3) | 0 (0.0) | 1 (4.5) |  |
|  | Moderate |  | 5 (16.7) | 1 (12.5) | 4 (18.2) |  |
|  | Severe |  | 24 (80.0) | 7 (87.5) | 17 (77.3) |  |
| Worst severity: Hand Eczema (%) |  | 87 |  |  |  | NaN |
|  | Clear |  | 0 (0.0) | 0 (0.0) | 0 (0.0) |  |
|  | Mild |  | 3 (3.4) | 0 (0.0) | 3 (4.1) |  |
|  | Moderate |  | 20 (23.0) | 2 (14.3) | 18 (24.7) |  |
|  | Severe |  | 64 (73.6) | 12 (85.7) | 52 (71.2) |  |
| Worst severity: Neurodermatitis (%) |  | 7 |  |  |  | NaN |
|  | Clear |  | 0 (0.0) | 0 (0.0) | 0 (0.0) |  |
|  | Mild |  | 0 (0.0) | 0 (0.0) | 0 (0.0) |  |
|  | Moderate |  | 2 (28.6) | 0 (0.0) | 2 (40.0) |  |
|  | Severe |  | 5 (71.4) | 1 (100.0) | 3 (60.0) |  |
| Worst severity: Nummular Eczema (%) |  | 25 |  |  |  | NaN |
|  | Clear |  | 0 (0.0) | 0 (0.0) | 0 (0.0) |  |
|  | Mild |  | 4 (16.0) | 1 (33.3) | 3 (13.6) |  |
|  | Moderate |  | 8 (32.0) | 1 (33.3) | 7 (31.8) |  |
|  | Severe |  | 13 (52.0) | 1 (33.3) | 12 (54.5) |  |
| Worst severity: Seborrheic Dermatitis (%) |  | 40 |  |  |  | NaN |
|  | Clear |  | 0 (0.0) | 0 (0.0) | 0 (0.0) |  |
|  | Mild |  | 3 (7.5) | 0 (0.0) | 3 (8.8) |  |
|  | Moderate |  | 16 (40.0) | 2 (40.0) | 13 (38.2) |  |
|  | Severe |  | 21 (52.5) | 3 (60.0) | 18 (52.9) |  |
| Worst severity: Stasis Dermatitis (%) |  | 3 |  |  |  | NaN |
|  | Clear |  | 0 (0.0) | 0 (NaN) | 0 (0.0) |  |
|  | Mild |  | 0 (0.0) | 0 (NaN) | 0 (0.0) |  |
|  | Moderate |  | 1 (33.3) | 0 (NaN) | 1 (33.3) |  |
|  | Severe |  | 2 (66.7) | 0 (NaN) | 2 (66.7) |  |
| Severity now: Atopic Dermatitis (%) |  | 222 |  |  |  | 0.841 |
|  | Clear |  | 13 (5.9) | 1 (3.7) | 12 (6.2) |  |
|  | Mild |  | 73 (32.9) | 10 (37.0) | 62 (32.3) |  |
|  | Moderate |  | 94 (42.3) | 10 (37.0) | 83 (43.2) |  |
|  | Severe |  | 42 (18.9) | 6 (22.2) | 35 (18.2) |  |
| Severity now: Contact Dermatitis (%) |  | 87 |  |  |  | 0.347 |
|  | Clear |  | 14 (16.1) | 1 (7.7) | 13 (17.6) |  |
|  | Mild |  | 31 (35.6) | 5 (38.5) | 26 (35.1) |  |
|  | Moderate |  | 33 (37.9) | 7 (53.8) | 26 (35.1) |  |
|  | Severe |  | 9 (10.3) | 0 (0.0) | 9 (12.2) |  |
| Severity now: Dyshidrotic Eczema (%) |  | 30 |  |  |  | 0.768 |
|  | Clear |  | 3 (10.0) | 1 (12.5) | 2 (9.1) |  |
|  | Mild |  | 13 (43.3) | 4 (50.0) | 9 (40.9) |  |
|  | Moderate |  | 8 (26.7) | 1 (12.5) | 7 (31.8) |  |
|  | Severe |  | 6 (20.0) | 2 (25.0) | 4 (18.2) |  |
| Severity now: Hand Eczema (%) |  | 87 |  |  |  | 0.329 |
|  | Clear |  | 2 (2.3) | 1 (7.1) | 1 (1.4) |  |
|  | Mild |  | 26 (29.9) | 6 (42.9) | 20 (27.4) |  |
|  | Moderate |  | 41 (47.1) | 5 (35.7) | 36 (49.3) |  |
|  | Severe |  | 18 (20.7) | 2 (14.3) | 16 (21.9) |  |
| Severity now: Neurodermatitis (%) |  | 7 |  |  |  | NaN |
|  | Clear |  | 0 (0.0) | 0 (0.0) | 0 (0.0) |  |
|  | Mild |  | 1 (14.3) | 0 (0.0) | 1 (20.0) |  |
|  | Moderate |  | 4 (57.1) | 1 (100.0) | 2 (40.0) |  |
|  | Severe |  | 2 (28.6) | 0 (0.0) | 2 (40.0) |  |
| Severity now: Nummular Eczema (%) |  | 25 |  |  |  | 0.499 |
|  | Clear |  | 5 (20.0) | 1 (33.3) | 4 (18.2) |  |
|  | Mild |  | 13 (52.0) | 1 (33.3) | 12 (54.5) |  |
|  | Moderate |  | 4 (16.0) | 0 (0.0) | 4 (18.2) |  |
|  | Severe |  | 3 (12.0) | 1 (33.3) | 2 (9.1) |  |
| Severity now: Seborrheic Dermatitis (%) |  | 40 |  |  |  | 0.465 |
|  | Clear |  | 7 (17.5) | 0 (0.0) | 7 (20.6) |  |
|  | Mild |  | 15 (37.5) | 3 (60.0) | 12 (35.3) |  |
|  | Moderate |  | 14 (35.0) | 1 (20.0) | 12 (35.3) |  |
|  | Severe |  | 4 (10.0) | 1 (20.0) | 3 (8.8) |  |
| Severity now: Stasis Dermatitis (%) |  | 3 |  |  |  | NaN |
|  | Clear |  | 0 (0.0) | 0 (NaN) | 0 (0.0) |  |
|  | Mild |  | 0 (0.0) | 0 (NaN) | 0 (0.0) |  |
|  | Moderate |  | 2 (66.7) | 0 (NaN) | 2 (66.7) |  |
|  | Severe |  | 1 (33.3) | 0 (NaN) | 1 (33.3) |  |
| Previous CT participation/attempts/consideration (%) |  | 281 |  |  |  | NaN |
|  | 1 |  | 0 (0.0) | 0 (0.0) | 0 (0.0) |  |
|  | 2 |  | 0 (0.0) | 0 (0.0) | 0 (0.0) |  |
|  | 3 or more |  | 0 (0.0) | 0 (0.0) | 0 (0.0) |  |
|  | None, but considered (and tried) |  | 0 (0.0) | 0 (0.0) | 0 (0.0) |  |
|  | None, but considered (never tried) |  | 0 (0.0) | 0 (0.0) | 0 (0.0) |  |
|  | None, never considered |  | 281 (100.0) | 34 (100.0) | 244 (100.0) |  |
| Worst severity ever (all types) (%) |  | 281 |  |  |  | NaN |
|  | Clear |  | 0 (0.0) | 0 (0.0) | 0 (0.0) |  |
|  | Mild |  | 4 (1.4) | 1 (2.9) | 3 (1.2) |  |
|  | Moderate |  | 64 (22.8) | 5 (14.7) | 59 (24.2) |  |
|  | Severe |  | 213 (75.8) | 28 (82.4) | 182 (74.6) |  |
| Worst severity now (all types) (%) |  | 281 |  |  |  | 0.441 |
|  | Clear |  | 13 (4.6) | 2 (5.9) | 11 (4.5) |  |
|  | Mild |  | 74 (26.3) | 9 (26.5) | 65 (26.6) |  |
|  | Moderate |  | 130 (46.3) | 12 (35.3) | 116 (47.5) |  |
|  | Severe |  | 64 (22.8) | 11 (32.4) | 52 (21.3) |  |

**Table S8. Response theme distributions for hardest to reach subgroup.**

| **Feature** |  | **Overall** | **In Hardest to Reach** | **Not in Hardest to Reach** | **p-value** |
| --- | --- | --- | --- | --- | --- |
| What are the reasons why you never participated in an eczema clinical trial? |  | 367 | 43 | 321 |  |
|  |  |  |  |  | 0.033 |
|  | Fear/risks/unknowns of trials | 43 (11.7) | 10 (23.3) | 33 (10.3) |  |
|  | Accessibility | 25 (6.8) | 6 (14.0) | 18 (5.6) |  |
|  | Aware | 162 (44.1) | 16 (37.2) | 144 (44.9) |  |
|  | Eligibility | 67 (18.3) | 5 (11.6) | 62 (19.3) |  |
|  | No interest | 70 (19.1) | 6 (14.0) | 64 (19.9) |  |
|  |  |  |  |  |  |
| What would motivate you to explore eczema clinical trials? |  | 369 | 27 | 339 |  |
|  |  |  |  |  | 0.836 |
|  | Burden of their disease | 161 (43.6) | 14 (51.9) | 146 (43.1) |  |
|  | Fear/risks/unknowns of trials | 48 (13.0) | 3 (11.1) | 45 (13.3) |  |
|  | Altruism | 22 (6.0) | 0 (0.0) | 22 (6.5) |  |
|  | Out of options | 79 (21.4) | 6 (22.2) | 72 (21.2) |  |
|  | Accessibility | 59 (16.0) | 4 (14.8) | 54 (15.9) |  |
|  |  |  |  |  |  |

**Figure S1. What motivated you to consider participating in an eczema clinical trial?**

987 major themes in responses for 636 respondents

**Figure S2. What were some of the issues getting in/enrolling in the eczema clinical trial?**

169 major themes in responses for 135 respondents

**Figure S3. What are the reasons why you never participated in an eczema clinical trial?**

367 major themes in responses for 281 respondents.

**Figure S4. What would motivate you to decide to explore eczema clinical trial opportunities?**

369 major themes in responses for 281 respondents
