# Supplementary figures and images for: Patient and caregiver motivators and barriers to eczema clinical trial participation: Analysis of survey data
Source: Skin Health Dis. 2023 Jun 22;4(5):e259. doi: 10.1002/ski2.259 (PMC11442076; doi:10.1002/ski2.259)

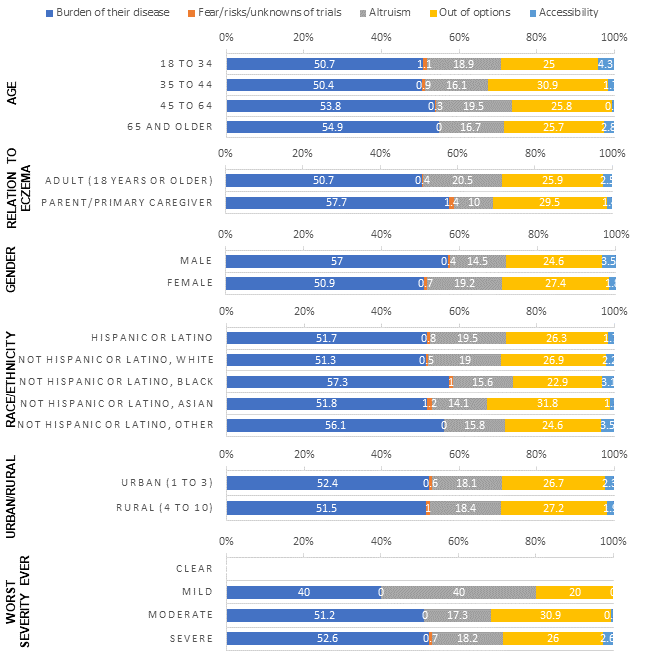

Supplement: Supplementary file 2 — Supporting Information S2 [file SKI2-4-e259-s004.PNG]

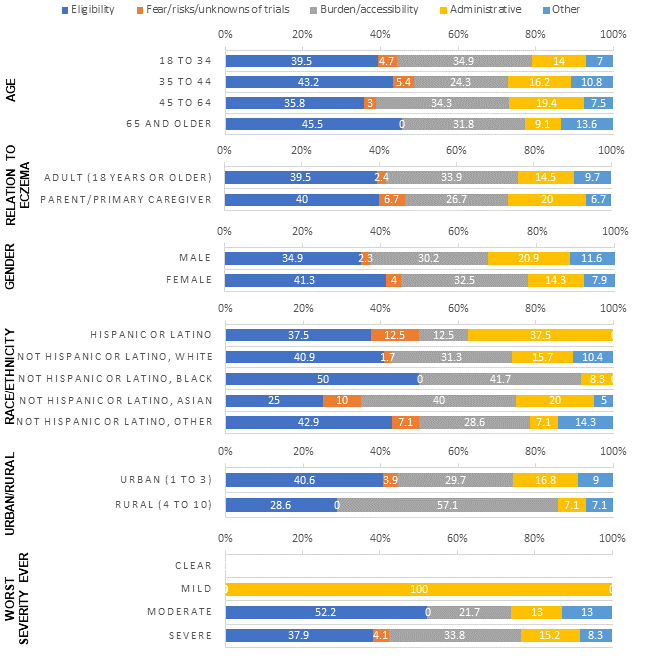

Supplement: Supplementary file 3 — Supporting Information S3 [file SKI2-4-e259-s003.PNG]

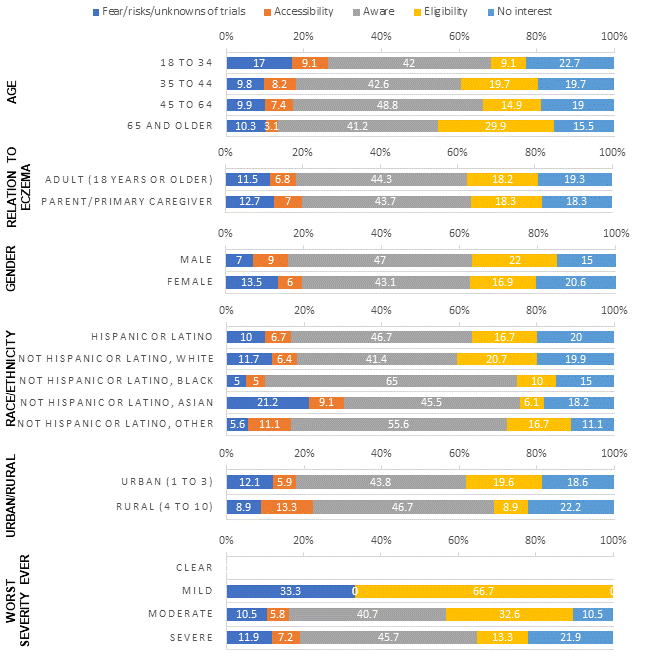

Supplement: Supplementary file 4 — Supporting Information S4 [file SKI2-4-e259-s002.PNG]

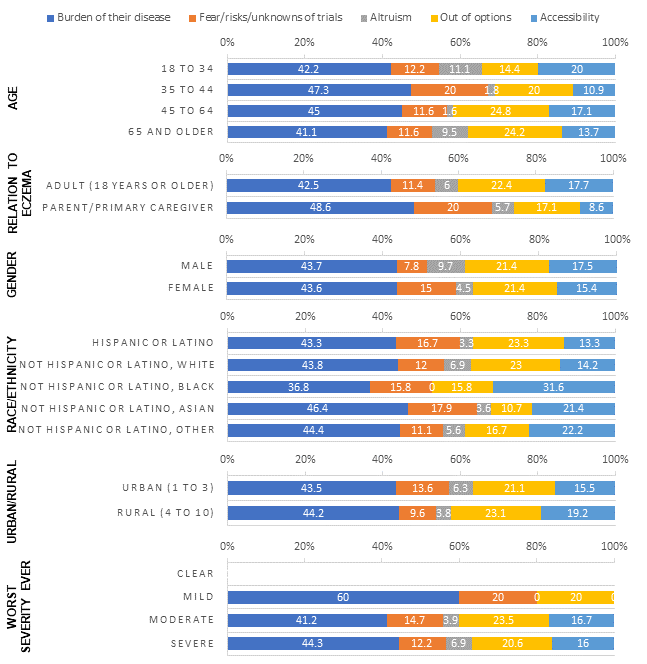

Supplement: Supplementary file 5 — Supporting Information S5 [file SKI2-4-e259-s005.PNG]
